# The Physician’s Visiting List

**Published:** 1891-01

**Authors:** 


### Book Reviews.

---

ATLANTA, GA., December 12, 1890.

THE PHYSICIAN'S VISITING LIST (LINDSY & BLAKISTON'S) FOR 1890. Published by P. Blakiston, Son & Co., Philadelphia.

For the active practitioner, there are few things more needed than a well-arranged visiting list to facilitate a proper record of a day's work. And of the many lists devised, none surpass the one published by P. Blakiston, Son & Co., of Philadelphia. Here are well-arranged, a list of officinal drugs, as well as those new and valuable ones, which as yet, have not been made officinal.

This little list contains also a well-arranged daily register, almanac, methods and suggestions, and valuable hints as to emergency calls, pathological rules, and an accurate table for calculating the period of utero gestation. Thus, one can readily see that for the moderate price of $1.25 or up to $2.00 and $3.00, according to the size and number of pages, Blakiston gives value received.

